# Advancing Obsessive–Compulsive Disorder Research: Insights from Transgenic Animal Models and Innovative Therapies

**DOI:** 10.3390/brainsci15010043

**Published:** 2025-01-04

**Authors:** Xinyuejia Huang, Linglong Xiao, Mengqi Wang, Yang Wu, Hao Deng, Wei Wang

**Affiliations:** Department of Neurosurgery, West China Hospital, Sichuan University, Chengdu 610041, China; hxyj2023@stu.scu.edu.cn (X.H.); xll2021@stu.scu.edu.cn (L.X.); wmq_1101@scu.edu.cn (M.W.); wuyang1@stu.scu.edu.cn (Y.W.); 2023324025260@stu.scu.edu.cn (H.D.)

**Keywords:** animal model, treatment, obsessive–compulsive disorder (OCD), transgenic, knockout (KO)

## Abstract

Obsessive–compulsive disorder (OCD) is a prevalent, chronic, and severe neuropsychiatric disorder that leads to illness-related disability. Despite the availability of several treatments, many OCD patients respond inadequately, because the underlying neural mechanisms remain unclear, necessitating the establishment of many animal models, particularly mouse models, to elucidate disease mechanisms and therapeutic strategies better. Although the development of animal models is ongoing, there remain many comprehensive summaries and updates in recent research, hampering efforts to develop novel treatments and enhance existing interventions. This review summarizes the phenotypes of several commonly used models and mechanistic insights from transgenic models of OCD, such as knockout mouse models. In addition, we present the advantages and limitations of these models and discuss their future in helping further understand the pathophysiology and advanced treatment. Here, we highlight current frontline treatment approaches for OCD, including neuromodulation and surgical interventions, and propose potential future directions. By studying gene mutations and observing phenotypes from available OCD animal models, researchers have classified the molecular signatures of each model reminiscent of changes in brain areas and neural pathways, with the hope of guiding the future selection of the most appropriate models for specific research in the OCD field.

## 1. Introduction

Obsessive–compulsive disorder (OCD) is a prevalent neuropsychiatric condition, with a lifetime prevalence estimated to range from 1% to 3% [1,2]. This prevalence remains consistent across various countries. During childhood and adolescence, about half of patients experience symptoms [3]. Moreover, nearly 90% of individuals with OCD also have comorbid psychiatric conditions, with anxiety disorders being the most prevalent [4]. The primary clinical features of this disorder include conscious compulsive behaviors and obsessive thoughts. Patients frequently encounter repetitive intrusive thoughts or behaviors, some of which may be meaningless or contrary to their intentions. These experiences result in distress, anxiety and aversion, and put a substantial burden on their families and society [5].

As the primary treatment option, OCD patients typically undergo cognitive–behavioral therapy (CBT) and receive medication, particularly, selective serotonin reuptake inhibitors (SSRIs) [6,7,8]. However, initially, these interventions yield limited efficacy, with almost 50% of OCD patients not responding adequately [9]. This underscores the urgent need to develop novel treatments or more efficacious disease-modifying therapies for OCD. Although gene therapy is a promising treatment approach, the identification of risk genes for OCD lags significantly behind that for other mental disorders [10,11], due in part to the lack of comprehensive whole-genome studies and the need for a deeper understanding of OCD’s neurobiological and genetic foundations [12].

Given the urgency for more efficient and complementary therapeutic strategies, the validation of animal models with translational relevance is crucial in the field [12]. In recent years, animal models, specifically mouse models, mainly because of their applications in gene mutation studies, have played a crucial role in genomics research. These models are important for exploring the complex pathophysiological basis of various diseases and elucidate the genetic and neural circuit mechanisms.

In OCD research, these models have shed light on previously obscure aspects of the disorder, thereby substantiating their critical importance. They are independent of both environmental influences and genetic variations [13], thus facilitating targeted exploration of the neural circuits associated with OCD. By parsing OCD-related behaviors, such as compulsions and anxiety, into specific neurobiological components, these models become invaluable tools for analysis. At the same time, they reveal molecular pathways associated with the cortico-striato-thalamo-cortical (CSTC) circuit—an area implicated in OCD. In addition, some relevant animal models have been corroborated by both genetic and imaging evidence, confirming their efficacy in investigating pathogenesis. This has led to the development of multiple investigative strategies using gene knockout animal models, significantly enriching our comprehension of the origins and progression of OCD, and potentially paving the way for innovative treatments for it and similar disorders.

Without attempting to review in detail all of the facets of OCD, we have summarized a few well-established and commonly used transgenic models, such as knockout (KO) models. For transgenic mouse models, *Hoxb8-KO*, *Slc1a1-KO*, *Sapap3-KO*, *Slitrk5-KO*, and *Spred2-KO* mouse models have been recently studied. We performed a meticulous comparison among the prevalent OCD mouse models, including phenotypic characterization and underlying mechanisms. In addition, advancements in neuromodulatory and surgical techniques, such as deep brain stimulation (DBS), have shown promise in addressing treatment-resistant OCD. By targeting some specific brain areas, these approaches help patients to go into remission and enhance the quality of life for affected individuals.

Given the interactions with genetic, neural, and environmental factors in OCD, researchers have focused on the future needs of developing novel model systems for OCD. Therefore, understanding the phenotypic characteristics and underlying mechanisms of OCD mouse models helps us translate findings from animal research to human clinical contexts, and contributes to diagnostic and therapeutic outcomes for OCD patients.

## 2. Biological Basis of OCD

### 2.1. Clinical Features of OCD

Obsessive–compulsive disorder (OCD), like other neuropsychiatric disorders, necessitates additional research to elucidate its underlying causes. OCD is primarily characterized by obsessions: intrusive, persistent, and unwanted thoughts or urges linked to heightened anxiety and compulsions, which are repetitive behaviors or mental acts often performed rigidly and ritualistically [14]. Generally, based on the CSTC circuit, a complex imbalance between the direct and indirect pathways can elucidate most OCD-related symptoms [15], and we will review the clinical manifestations of OCD and associated molecular mechanisms (Table 1). Contemporary research posits that various factors, encompassing genetics and the environment, exert intricate effects on the evolution of OCD [5]. For instance, there is evidence demonstrating that dopaminergic agents such as amphetamine (AMPH) and L-DOPA can trigger repetitive behaviors in OCD patients [16,17,18]. Family and twin studies show that OCD is a multifactorial condition strongly linked to genetic factors [14]. Furthermore, these studies indicate a genetic predisposition to OCD, with the identification of genes correlated with the disorder, such as glutamatergic-related genes in humans (such as *PTPRD*, *GRID2*, *DLGAP1*, and *DLGAP3 (SAPAP3)*), alongside genes consistently affecting neurotransmission (such as *GRIN2B* and *SLC1A1*) [19,20]. Some see that the interaction between the brain and environment and neuroplasticity might modulate the compulsive-like behavior related to protein regulation [21]. To summarize, we all know that the origin of OCD is complex and shaped by a nexus of genetic and environmental determinants. Our understanding of the disorder can be enhanced by continuing investigation, leading to more efficacious treatment modalities.

Although we cannot fully understand the pathogenesis of OCD, the hypothesis that it involves several interrelated mechanisms is proposed: cognitive–motional abnormalities, neural circuitry dysregulation, functional and structural neural alterations, and molecular irregularities.

### 2.2. Current Research on Animal Models of OCD

The genetic basis of OCD is multifactorial and polygenic, presenting challenges in identifying a single gene correlation. Analysis of genetic associations in thousands of cases has shown that approximately 85% of these associations have odds ratios of less than 1.5 [30]. Due to OCD’s complexity, translating biological knowledge into practical applications such as clinical trials can be daunting. Therefore, the use of animal models for large-scale genetic studies is essential. Some researches enable the exploration of numerous hypothesized genetic factors, revealing their functional relevance and ultimately contributing to the development of enhanced diagnostic and therapeutic approaches for OCD [31]. For the last few years, our research has been dedicated to developing dependable animal models for OCD and using these models to look into the mechanisms of the disorder and evaluate the efficacy of new treatments. Although primarily utilizing rodents, these models only partially replicate the complexities of OCD but demonstrate validity to some extent.

There are currently three primary types of animal models for OCD: genetic models, pharmacological models, and behavioral models. Genetic models involve limited research but can replicate genetic changes found in patients, furthering the understanding of disease mechanisms and demonstrating good face validity. However, these models cannot fully meet all established validity criteria [32], such as the *Slc1a1* and *Sapap3* models, because such models primarily exhibit face validity since no single gene knockout has been definitively linked to OCD in humans. Pharmacological models induce OCD-like behaviors using drugs and are essential for drug testing, albeit with potential face and construct validity. Pharmacological models that induce stereotyped behavior have been documented in the literature. Scheel-Krüger et al. reported that the specific GABA agonist muscimol elicited strong amphetamine-like stimulation and stereotyped behavior following intracranial injection [33]. Additionally, glutamate receptor agonists, such as NMDA agonists, are known to induce stereotyped behavior [34]. Furthermore, administration of apomorphine has been shown to activate dopamine receptors in the neostriatum and to induce stereotypical behavior in numerous studies across various species [35,36]. Moreover, pharmacological stimulation of postsynaptic serotonin receptors can also induce stereotyped behavior, which is associated with hypoactivity in serotonin pathways [33,37]. Behavioral models are suitable for situations where compulsive behaviors arise spontaneously. While the behavioral models demonstrate good face validity, they do not consider the genetic information underlying the origin of OCD.

Finding effective animal models of OCD needs critical evaluation rather than blindly trusting study conclusions. Animal models have the advantage of the ability to perform repetitive procedures, which are not easily accessible in clinical experiments. But it is controversial for us to use these models, due to the translation of these models into clinical practice. Preclinical operations, such as genetic, pharmacological, network, or other approaches, in animal models provide the opportunity to obtain high-quality and biologically grounded data for studying compulsive behaviors. It is important to note that implementing environmental control methods in a clinical research setting is impractical [5]. So, we will review several transgenic models that have provided intriguing findings related to anxiety-like behavior and unusual repetitive and compulsive behaviors.

### 2.3. Transgenic Mouse Models of OCD

In recent years, we have carried out extensive research on the genetic basis of OCD. This research necessitates significant time and resources and currently should focus on translating genetic discoveries into practical applications. However, it is challenging to demonstrate relevant cognitive constructs as they may apply to these mouse models [38]. Knockout mouse models enable researchers to effectively study genes associated with OCD and assess their impact on behavior and the nervous system, with the aim to gain a deeper understanding of the underlying mechanisms of OCD and provide a theoretical foundation for the development of new therapies.

Additionally, the use of transgenic models has resulted in significant findings in investigating OCD mechanisms. While transgenic models are commonly used for various scientific purposes, they have proven valuable in exploring potential mechanisms of OCD-related behaviors. In the following sections, we will review several transgenic models that have currently provided intriguing findings related to anxiety-like behaviors as well as unusual repetitive and compulsive behaviors, such as compulsive grooming behavior, perseverative responses in reverse learning processes, and perseveration in excessive locomotion behaviors (Table 2).

#### 2.3.1. Hoxb8-Knockout Mouse

*HOXB8* is a component of the mammalian *Hox* complex, which consists of 39 transcription factors that govern anterior–posterior axis patterning. In a study, a *Hoxb8*-knockout mouse model was established to explore the potential mechanisms associated with compulsive grooming behavior in OCD patients [39]. The inactivation or mutation of the *Hoxb8* gene leads to persistent anxiety, OCD-like behaviors, and prolonged self-grooming in mice, resulting in extensive hair loss and severe skin damage [40].

Importantly, we have to know that *Hoxb8*, despite its transcription factor nature, plays a crucial role in the development of multicellular organisms by accurate reflections of structural changes. Although the *Hoxb8* mutant mouse model did not exhibit morphological changes, it showed noticeable behavioral alterations [39]. Furthermore, studies have indicated that *Hoxb8* is expressed not in neurons but in a specific subset of cells called microglia, which originate from myeloid progenitors in the brain [41]. As a result, the knockout of the *Hoxb8* gene resulted in a 15% reduction in the overall population of microglia in a mouse model [42].

The transplantation of bone marrow from wild-type mice into *Hoxb8*-knockout mice produced some very interesting effects. This process not only stimulates the proliferation of donor wild-type microglia in the brains of recipient *Hoxb8* gene knockout mice, but also significantly improves their abnormal behaviors [42]. These findings suggest a causal link between the reduction in this specific subset of microglia and the occurrence of excessive grooming behavior.

The expression of the *Hoxb8* gene varies among different species. *Hoxb8* is widely expressed in mouse central nervous system, including the olfactory bulb, brainstem, hippocampus, frontal cortex, and caudate–putamen. Conversely, in humans, *Hoxb8* is primarily expressed in the brainstem, olfactory bulb, cortex, and striatum [39,42], with a particular emphasis on cortical regions associated with the mechanisms of OCD, such as the orbital frontal cortex and anterior cingulate cortex [12].

Nagarajan and colleagues revealed that optogenetic stimulation of *Hoxb8* microglia in specific brain regions can trigger anxiety-like and self-grooming behaviors [40]. Additionally, they demonstrated that by inducing the early response of the c-Fos protein and optogenetic stimulation of *Hoxb8* microglia in vitro, these microglia can activate neighboring neural activity and induce behaviors akin to OCD. These findings imply that *Hoxb8* microglia likely play a significant role in eliciting these behaviors [40]. Furthermore, these studies reveal an association between the *Hoxb8* gene, the immune system, and OCD, improving new potential treatment for OCD by examining the involvement of *Hoxb8* microglia in the development of excessive grooming behavior.

#### 2.3.2. Slc1a1-Knockout Mouse

The *SLC1A1* gene, located on chromosome 9p24, is expressed in brain regions associated with OCD, such as the cerebral cortex, striatum, and thalamus [43]. As research on the genetic mechanisms associated with OCD has increased, more studies have focused on the role of the glutamate transporter gene *SLC1A1/EAAC1* in pathogenesis and proposed mechanisms [44,45,46,47]. This gene primarily encodes excitatory amino acid transporter-3 (EAAT3), which plays a crucial role in neurotransmission in mammals, affecting approximately 30–40% of synapses in the mammalian brain [48]. Notably, the alleles of *SLC1A1* most strongly associated with OCD lead to an increase in the levels of EAAT3 protein [44].

This study is in line with the initial findings of Zike et al. [25], indicating that the conditional overexpression of EAAT3 in the forebrain (EAAT3glo/CMKII mice, including the frontal cortex, hippocampus, and striatum) leads to an increase in specific OCD-related behaviors. These include heightened grooming and anxiety-related behaviors observed in open field, light–dark box, and marble-burying tests [49].

Furthermore, Gonzalez et al. [50] demonstrated that reducing EAAT3 can prevent drug-induced repetitive behaviors, suggesting that reducing EAAT3 is a potential therapeutic target for OCD. However, studies on EAAT3 heterozygous mice revealed that despite reduced expression of the *Slc1a1* gene, there was no impact on anxiety-related or compulsive behaviors or neurotransmitter levels in the cortico-striatal circuit [50]. Nevertheless, inducing the overexpression of this gene in the striatum led to an increase in repetitive or stereotyped behaviors in mice [25]. Therefore, it may be necessary to block the activity of this transporter protein for effective treatment.

Bellini et al. reported that mice deficient in EAAT3 exhibited heightened anxiety-like behaviors and aberrant grooming patterns [51]. This led to the elucidation of a new molecular mechanism whereby Eaac1 moderates the activity of metabotropic glutamate receptor 1 (mGluR1) within the striatum, in turn enhancing the expression of D1 dopamine receptors (D1Rs) and modulating repetitive movements. Furthermore, EAAT3 deficiency disrupts the processes of synaptic excitatory transmission. These data indicate that *SLC1A1* gene variants may be implicated in the glutamatergic dysregulation observed in OCD patients [52], shedding light on the molecular underpinnings and informing the creation of targeted therapies.

#### 2.3.3. Sapap3-Knockout Mouse

The *SAPAP* (SAP90/PSD-95-associated protein, also called discs-large-associated proteins (DLGAPs)) family is composed of crucial postsynaptic scaffold proteins that regulate the trafficking of AMPA- and NMDA-type glutamate receptors to the postsynaptic membrane of excitatory synapse density proteins [53]. *Sapap3* (also known as *Dlgap3*) is highly expressed in the mouse striatum and enriched within specific astrocyte subcompartments to modulate actin cytoskeleton organization [54,55]. While studying cellular and neural circuit mechanisms in human subjects is challenging, transgenic animal models can be instrumental in demonstrating alterations in the cortico-striatal circuit activity that contribute to compulsive behavior. This discussion will centre on the utilization of *Sapap3*-knockout mouse models in research related to OCD.

The *SAPAP* protein interacts with the GK domain of postsynaptic density protein-95 (PSD-95), also known as SAP-90 or synapse-associated protein 90. It is a member of the membrane-associated guanylate kinase (MAGUK) family. PSD-95 and PSD-93 form a synaptic scaffold at postsynaptic sites [5], promoting the clustering of ion channels and crucially anchoring the molecular structure of NMDA/AMPA receptor channels [56]. Clinical genetic studies have established a connection between allelic gene variations in *SAPAP1* and *SAPAP3* and OCD [57,58]. In addition, researchers conducted an initial investigation into the distribution of *Sapap* family proteins in the mouse brain [54], revealing predominant dendritic expression of *Sapap3* in the hippocampus, striatum, and cortex, with the highest expression observed in the striatum. These findings strongly suggest a potentially significant association between *Sapap3* and OCD, providing a basis for utilizing *Sapap3*-knockout mouse models to study compulsive behavior in rodents.

*Sapap3*-knockout mice are widely used and extensively studied mouse models for OCD research. Because they lack the postsynaptic scaffolding protein SAP90/PSD95-associated protein 3 (*Sapap3*) [54,59], these mice exhibit similarity with human OCD symptomatology in many ways, such as anxiety-like behavior, increased self-grooming, and impaired habit learning [53,60,61,62]. Additionally, they show reduced motor abilities (unrelated to grooming) and decreased average body weight [61,63]. Treatment with SSRIs has been shown to alleviate many of these symptoms, and research has established an association with the overactive cortico-striato-thalamo-cortical (CSTC) circuits [53,64,65]. Manning et al. observed an operant reversal of learning impairment in a *Sapap3*-knockout mouse model, which was linked to reduced activity in the medial prefrontal cortex (mPFC) [66]. Furthermore, recent research has revealed that the deletion of *Sapap3* leads to pathway-specific and non-synaptic changes in the functionality of circuits within the dorsolateral striatum [67], suggesting that a series of pathway-specific and intracellular functional changes in this *Sapap3*-KO model may play a key role in the pathogenesis of OCD.

In general, although *DLGAP3/SAPAP3* has not shown findings from genome-wide association studies (GWAS) in OCD research, previous research has confirmed the efficacy of this animal model and established a reliable theoretical foundation for its potential clinical relevance in the treatment of OCD.

#### 2.3.4. Slitrk5-Knockout Mouse

*Slitrk5*-knockout mouse models are widely utilized as transgenic animal models for investigating central nervous system functions. Its primary function is to encode a transmembrane protein, one of six members of the *SLITRK* family. This gene is broadly expressed within the central nervous system and plays a crucial role in various processes, including neuronal differentiation, dendritic branching, synaptic growth, synaptogenesis, and signal transduction, among others [68].

The *Slitrk* family gained prominence following its discovery through differential gene expression screening in mice with neural tube defects in 2001. Subsequent studies confirmed the presence of all six members of the *SLITRK* family. These genes are located on chromosome 13 (*SLITRK1*, *SLITRK5*, and *SLITRK6*), chromosome 3 (*SLITRK3*), and the X chromosome (*SLITRK2* and *SLITRK4*) [69]. These proteins are primarily expressed within the developing central nervous system [70,71]. In addition, their expression includes various cell types, such as brain tumors, lymphomas, embryonic stem cells, and hematopoietic stem cells, with variations observed among different family members [72].

*Slitrk5* expression is prominent in specific regions, including the CA1 region of the hippocampus [73], the frontal lobe of the brain, the spinal cord, and the medulla. Moreover, *Slitrk1* is expressed in mature neurons, *Slitrk2* is highly expressed in the ventricular layer of the brain, and *Slitrk6* is regionally expressed in the diencephalon. Emerging research suggests potential associations between *Slitrk5* and the pathogenesis of various central nervous system disorders, including OCD [74]. Additionally, studies indicate that manipulating *Slitrk5* expression may facilitate the restoration of neural system function, making it a potential therapeutic target for central nervous system disorders.

The establishment of a *Slitrk5*-knockout mouse model is essential for understanding the mechanisms underlying central nervous system development and disorders. Initially, these mice displayed heightened anxiety-like behavior, as assessed through the elevated plus maze, open field, and novel object tests. This behavior is subsequently followed by repetitive and excessive self-grooming, leading to severe facial skin injury and hair loss [74]. These behavioral characteristics closely resemble the phenotype observed in the *Sapap3*-knockout mouse model [53]. Both models exhibit cortico-striatal abnormalities and show symptom relief after fluoxetine treatment. In conclusion, the use of the *Slitrk5*-knockout mouse model provides a valuable tool for investigating the link between human genes and OCD, exploring its pathogenesis, and identifying potential targets for targeted interventions.

#### 2.3.5. Spred2-Knockout Mouse

A study conducted in 2018 investigated the effects of *Spred2*-knockout in mice. The findings demonstrated that these mice exhibited behaviors indicative of OCD and anxiety, which closely resembled those observed in mice lacking the *Sapap3* gene [75]. Sprouty-related enabled/vasodilator-stimulated phosphoprotein homology 1 (EVH1) domain-containing 2 (*Spred2*) inhibits the mitogen-activated protein kinase (MAPK)/extracellular signal-regulated kinase (ERK) pathway and promotes autophagy in several cancers [76]. This pathway is crucial for controlling various cellular processes, including cell proliferation and differentiation. Despite the understanding of the influence of the *Spred* family on the Ras/ERK-MAPK pathway, the specific mechanism by which it operates remains incompletely understood. The *Spred* family comprises three members: *Spred1*, *Spred2*, and *Spred3*. These proteins possess an EVH-1 domain at their N-terminus, a c-KIT binding domain, an SPR domain at their C-terminus, and two uncharacterized domains [77,78].

The *Spred2* gene encodes a key regulatory factor protein that oversees the Ras/ERK-MAPK pathway, a cellular cascade activated by brain-derived neurotrophic factor (BDNF). In the *Spred2*-knockout mouse model, researchers observed changes in neural transmission within the thalamus–amygdala circuit. Furthermore, subsequent research revealed that the *Spred2*-knockout mouse model exhibited changes similar to those observed in *Sapap3*-knockout mice, and these changes appeared to be age-related [79]. These changes indicate developmental irregularities, suggesting that *Spred2* is involved in the development of the CNS in mice [80].

Researchers also find that changes in the *Spred2* and *Sapap3* genes are linked to functional alterations in the prefrontal cortex, striatum, and other brain regions. The consistency of these in both rodents and humans indicates the conservation of genetic mechanisms associated with OCD. Currently, there is limited research on the role of the *Spred2* gene in OCD. And the similarities between *Sapap3* and *Spred2* warrant further investigation into the potential relevance of the *Spred2* gene to OCD. Overall, these investigations may significantly contribute to a deeper understanding of OCD development and provide new insights into the neural circuitry underlying OCD. Below, we provide a concise comparison between humans and corresponding mouse gene-knockout models (Table 3).

## 3. Treatment Strategies for OCD

### 3.1. Psychotherapy and Pharmacotherapy

The recommended first-line treatment for OCD is CBT, which includes exposure and response prevention (ERP), as well as pharmacological interventions such as SSRIs [7,86]. Current treatment guidelines in the United States endorse three first-line treatments for OCD (SSRI, CBT, and SSRI + CBT), and propose combination therapy for patients with an inadequate response to single treatments or those with treatment-resistant OCD [87]. CBT, which focuses on understanding the symptoms and treatment process of OCD, involves the formation and elimination of patient fears [88]. Although ERP specifically targets this process, it remains uncertain whether ERP can potentially influence the pathophysiology of OCD [89].

For patients with severe OCD or those unable to comply with CBT requirements, the APA guidelines recommend using SSRIs alone. Some patients have a slow response to treatment, creating potential bias with short-term SSRI therapy [87,90]. The guidelines suggest a minimum duration of 8–12 weeks for SSRI treatment (initially at the maximum tolerated dose for 4–6 weeks) before considering a change in medication strategy. If CBT is not feasible, the use of SSRIs alone is also warranted. Combination therapy with both SSRIs and CBT is an option when patients respond partially to monotherapy or show an SSRI response. A preponderance of evidence supports this combination therapy and demonstrates symptom improvement with these interventions [90,91]. Nevertheless, about two-thirds of OCD patients do not achieve satisfactory outcomes, likely due to adverse drug reactions or poor patient adherence [92,93,94]. In addition, transgenic animal models are valuable for developing and screening anti-compulsive drugs because of their strong validity and capacity to quickly produce large, phenotypically stable cohorts [95].

### 3.2. Neuromodulation and Neurosurgery

Approximately 20–25% of individuals diagnosed with OCD exhibit resistance to conventional pharmacological and psychological treatments [96]. Therefore, surgical and neuromodulatory treatments are becoming promising alternatives. These methods aim to influence the CSTC circuit, which is pivotal in OCD pathology. Neuromodulation includes invasive and noninvasive techniques, such as stereotactic ablation, transcranial direct current stimulation (tDCS), repetitive transcranial magnetic stimulation (rTMS), and deep brain stimulation (DBS) [96]. With recent progress in OCD-related circuitry, these findings may offer therapeutic potential. Over the past decade, neuromodulation strategies have emerged as a promising treatment for psychiatric disorders by targeting circuit function [95]; for example, as a non-invasive brain stimulation treatment for these disorders, the therapeutic effects of tDCS can be maintained for some time [97]. Surgical approaches, in particular, show greater efficacy in OCD management than in other psychiatric conditions but are considered for patients who are unresponsive to traditional treatments [10,98]. Furthermore, optogenetic activation of the lateral orbitofrontal cortex (LOFC)–striatal circuit restored fast-spiking neuron microcircuits, reducing compulsive grooming in *Sapap3*-KO mice and suggesting a potential therapeutic target in OCD [64]. Additionally, circuit manipulation tools, like optogenetics and chemogenetics in animal models, have advanced our understanding of how altered circuits cause maladaptive behaviors [95].

**Figure 1:**
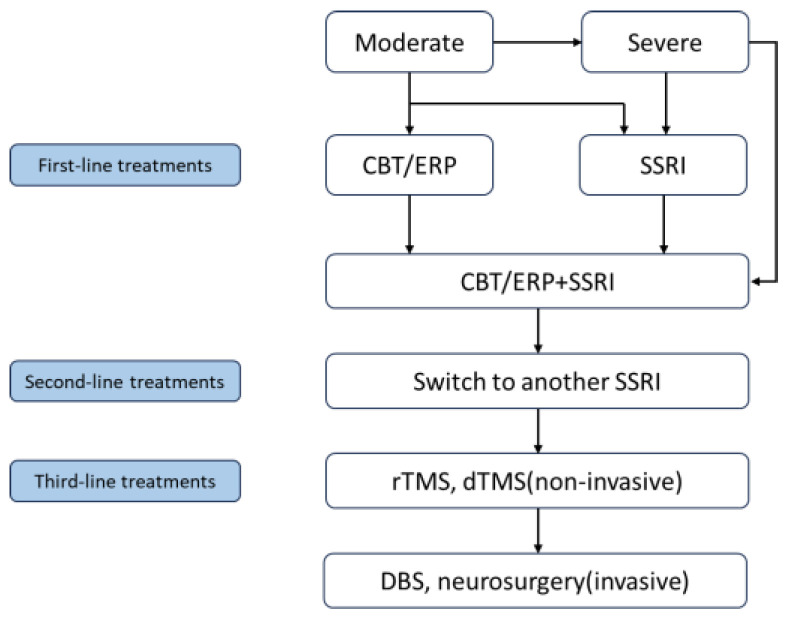
A Treatment algorithm for OCD patients. Note. CBT, cognitive behavioral therapy; ERP, exposure and response prevention; SSRI, selective serotonin reuptake inhibitor; rTMS, repetitive transcranial magnetic stimulation; dTMS, deep transcranial magnetic stimulation; DBS, deep brain stimulation.

“Traditional” neurosurgical procedures, such as stereotactic ablation, involve irreversible focal tissue ablation in specific regions of the CSTC circuit. However, these procedures can lead to adverse reactions, including nausea, vomiting, seizures, and cognitive decline. In contrast, DBS offers reversibility and adjustability and is primarily used to treat movement disorders, such as Parkinson’s disease and essential tremors [99,100]. Potential targets for DBS in OCD treatment include the striatal regions, such as the anterior limb of the internal capsule (ALIC)/nucleus accumbens or the subthalamic nucleus (STN) [101]. Recent studies have indicated that DBS is equally effective as ablative surgery [102,103]. However, its usage is recommended for long-term (more than 5 years) patients with OCD [96]. In this context, circuit manipulation in animal models may help optimize DBS protocols for OCD by selecting precise stimulation sites and targeting specific circuit alterations [104]. Although DBS may prove an effective treatment for OCD patients [105], there is currently no consensus on the stimulation site, frequency, intensity, treatment duration, or need for maintenance of each technique. Moreover, no comprehensive review of alternative brain stimulation methods for treating treatment-resistant OCD has been conducted. Further research is required to explore the underlying mechanisms of OCD and identify specific treatment targets. The treatment algorithm for OCD patients is summarized in Figure 1.

## 4. Discussion

Recent advancements have helped the development of various gene-knockout animal models that exhibit behaviors similar to those observed in OCD patients, such as repetitive self-grooming and anxiety-like behaviors. These models offer valuable insights into OCD, yet their utility is tempered by species-specific limitations. We need to acknowledge that these existing models do not fully encapsulate the multifaceted clinical manifestations of OCD, highlighting the need for further refinement in their application. Despite these questions, gene knockout models are pivotal in circumventing constraints inherent in human studies—such as environmental and genetic background variability—enabling a more subtlety understanding of the neurobiological mechanisms underpinning gene-related phenotypes, so this advances OCD medication research. However, the reliance on overexpression in these models, which results in behaviors analogous to OCD, limits their capacity to fully mirror the human condition. Consequently, there is a pressing need for innovative research models that more accurately reflect the complex nature of human OCD.

Current transgenic animal models are possibly underdeveloped for OCD research, and some lack strong theoretical underpinning, but they pave the way for innovative research avenues, ideas, and methods. These models facilitate an in-depth examination of the connections among genes, pathways, and circuits, providing us with an effective strategy to refine the modeling process and present a straightforward route for forthcoming OCD studies.

Additionally, in the treatment of OCD, there is growing evidence that neuromodulation and surgical interventions aiming at CSTC circuitry are both safe and effective and are recommended in several clinical guidelines and expert consensuses. DBS and minimally invasive ablative surgeries present potential long-term solutions for refractory OCD, provided that they adhere to treatment guidelines. Advanced research techniques can help us gain an improved understanding of the fundamental pathophysiology of OCD, such as neuroimaging, electrophysiology, comprehensive data analysis, and integrated behavioral models. This is expected to lead to the creation of customized treatment approaches for those affected, marking a significant step forward in the personalized management of OCD.

In summary, this review offers an overview of several frequently used OCD transgenic animal models, including knockout mouse models, and discusses their potential future in OCD research. More importantly, our review updates the literature with the most recent findings on current cutting-edge therapies and treatments for OCD (Table 4), encompassing neuromodulation and surgical interventions. Future directions for advancing more effective therapies, particularly for patients resistant to traditional therapeutic methods, are also highlighted. And there is a need in the realm to develop more diverse transgenic models to explore the mechanisms of OCD. Compared with the changes in the brains of patients and genetic findings in animal models, we aim to find a better selection of the most appropriate models for relevant research questions and help guide future translational research.

By comparing genetic findings in these models with observed changes in the brains of individuals with OCD, we aim to better inform the selection of the most appropriate models for relevant research questions and help to guide future translational studies.

## 5. Conclusions

Transgenic animal models, such as knockout mice, help us make deeper insights into the disorder’s pathophysiology and develop current therapies. Our findings also highlight the potential of advanced treatment modalities, including neuromodulation and neurosurgery (e.g., deep brain stimulation). Because DBS has had emerging success in the treatment of movement disorders, such as Parkinson’s disease, essential tremor, and so on, the usage of these therapies has shown promise in challenging pathologies of different psychiatric disorders. As we are researchers deeply committed to advancing the field, this study aims to provide a comprehensive and rigorous analysis that serves as a foundation for future work. And a multitude of different animal models are under development, and more time will be required to differentiate an optimal path forward for the field.

## Figures and Tables

**Table 1:** Clinical features and related mechanisms.

Clinical Feature	Molecular Mechanisms	Neural Circuits Involved
Obsessions (Intrusive Thoughts)	Dysregulation of serotonin transporter genes (e.g., SLC6A4) [22]	CSTC circuit (OFC, caudate nucleus) [23]
Compulsions (Repetitive Behaviors)	Alterations in glutamate receptor genes (e.g., GRIN2B and *SLC1A1*) [24,25]	CSTC circuit (ACC, dorsolateral striatum) [26]
Emotional Dysregulation (Anxiety)	Elevated cortisol due to HPA axis dysfunction [27,28]	Limbic system (amygdala) [29], vmPFC

Note. SLC6A4, serotonin transporter, 5-HTT, or SERT; OFC, orbitofrontal cortex; PFC, prefrontal cortex; vmPFC, ventromedial PFC; ACC, anterior cingulate cortex; HPA, hypothalamic–pituitary–adrenal.

**Table 2:** Animal models of OCD and related disorders.

Model	Phenotype	Putative Mechanism
Genetic		
*Hoxb8* knockout mouse	Excessive self-grooming and anxiety	Neuronal development, immune involvement
*SLcla1* knockout mouse	Decreased self-grooming and AMPH response	Decreased AMPH-induced c-Fos, D1R density, and basal extracellular dopamine in DS
*Sapap3* knockout mouse	Excessive self-grooming, anxiety, and skin lesions	Striatal neuronal differentiation and neurotransmission
*Slitrk5* knockout mouse	Excessive self-grooming, anxiety	Postsynaptic scaffolding protein at excitatory synapses highly expressed in the striatum
*Spred2* knockout mouse	Excessive self-grooming, anxiety	Increased EPSCs in DLS and LA Putative release of inhibition of Ras/ERK-MAPK signaling pathway

Note. AMPH, amphetamine; D1R, D1 dopamine receptor; DS, dorsal striatum; EPSCs, excitatory postsynaptic current; DLS, dorsolateral striatum; LA, lateral amygdala.

**Table 3:** Comparison of findings between humans and mouse models.

Gene	Defects in OCD	Mouse Model	Phenotype	Comparison
*HOXB8*	Linked to OCD-like behavior and anxiety [81]	*Hoxb8*-KO mouse	Excessive self-grooming, anxiety	Similar repetitive behaviors, but mice lack the full spectrum of human obsessions.
*SLC1A1*	Affecting glutamate uptake transporter [16]	*SLcla1*-KO mouse	Decreased self-grooming and AMPH response	Similar glutamate dysregulation, but also related to human TS [82].
*SAPAP3*	Synaptic protein mutations linked to OCD	*Sapap3*-KO mouse	Excessive grooming, anxiety, and skin lesions	Phenotype matches with human OCD symptomatology [62].
*SLITRK5*	Implicated in synapse formation abnormalities [83]	*Slitrk5*-KO mouse	Excessive self-grooming, anxiety	Impair synaptogenic activity [84]; in humans, symptoms are more diverse.
*SPRED2*	Dysregulation of Ras/MAPK pathway	*Spred2*-KO mouse	Excessive self-grooming, anxiety	Behavioral overlap; additional impacts in humans remain unexplored [85].

Note. TS, Tourette Syndrome.

**Table 4:** Summary of mechanisms, benefits, and limitations of current therapies for OCD.

Therapy	Mechanism	Benefits	Limitations
CBT	Cognitive restructuring	Non-invasive	Requires patient compliance
SSRIs	Serotonin reuptake inhibition	Effective for many patients	Limited efficacy in some cases
tDCS	Modulate brain activity	Portable and low-cost	Early-stage research
Stereotactic Ablation	Irreversible lesioning of specific brain regions	Effective for highly treatment	Risk of cognitive side effects
DBS	Targeted brain stimulation	Reversible, adjustable	Expensive, requires surgery

Notes: CBT and SSRIs are recommended first-line treatments. Neuromodulation techniques such as tDCS and DBS are particularly promising for treatment-resistant OCD.
